# Demographic, laboratory findings and diagnostic evaluation among high risk patients with mucopolysaccharidosis in Malaysia

**DOI:** 10.1016/j.dib.2019.104377

**Published:** 2019-08-09

**Authors:** Affandi Omar, Julaina A. Jalil, Norashareena M. Shakrin, Lock H. Ngu, Zabedah M. Yunus

**Affiliations:** a Biochemistry Unit, Specialised Diagnostic Centre, Institute for Medical Research, Ministry of Health Malaysia, Jalan Pahang, 50588, Kuala Lumpur, Malaysia; b Genetic Department, Kuala Lumpur Hospital, Ministry of Health Malaysia, Jalan Pahang, 50586, Kuala Lumpur, Malaysia

## Abstract

This article contains information related to a recent study “Selective screening for detection of mucopolysaccharidoses (MPS) in Malaysia; A Two-year Study” Affandi et al., 2019. Any patient registered under government healthcare facilities in Malaysia and fit at least two inclusion criteria were included in this selective screening. Urine and blood from these high risk patients were obtained and analysed for glycosaminoglycans (GAGs) level before characterization using high resolution electrophoresis (HRE). Thereafter, enzyme assay for different types of MPS based on result of HRE were determined using specific substrate. Demographic data as well as laboratory findings were tabulated and analysed. The data of this study demonstrate between clinical presentation and laboratory findings among high risk patients of MPS and can be employed to improve diagnosis of MPS.

**Table:** Specifications Table

Subject area	*Biochemical Genetics*
More specific subject area	*Inborn Errors of Metabolism*
Type of data	*Table*
How data was acquired	*Tecan Fluorometer Infinite M200 (2010), High resolution of electrophoresis*
Data format	*Raw, Analysed data*
Experimental factors	*Selective screening among high risk patients of mucopolysaccharidoses (MPS) in Malaysia based on inclusion criteria*
Experimental features	*Urine and blood from patients with high risk symptoms of mucopolysaccharidoses were analysed using test method. Demographic data and obtained results were analysed*
Data source location	*Kuala Lumpur, Malaysia*
Data accessibility	*The dataset is accessible within this article data*
Related research article	*Affandi Omar,* Julaina A.Jalil*,* Norashareena M.Shakrin*,* Lock H.Ngu*,* Zabedah M.Yunus*. Selective screening for detection of mucopolysaccharidoses in Malaysia; A two-year study (2014–2016). Molecular Genetics and Metabolism Reports 19 (2019) 100469*

**Table:** 

**Value of the data** • The dataset in this article will help further research on inborn error of metabolism in Malaysia. Policy maker, researchers and other stakeholders can use the dataset to compare the findings whether to include the commonest type of MPS in LSD newborn screening in future. • The dataset provides demographic data of patients with high risk of mucopolysaccharidoses • Data in this work provides further understanding of the correlation between clinical presentation and laboratory findings in patients with high risk of MPS.

## 1 Data

This article presents data from high-risk patients profiling of mucopolysaccharidoses in Malaysia from 2014 to 2016 related to the recent study described by Omar A. [1]. Mucopolysaccharidoses (MPS) is a group of disorders characterised by the accumulation of glycosaminosglycans (GAGs) in tissues and organs due to defects in specific enzymes contained within lysosomes. Chondroitin sulphate (CS), dermatan sulphate (DS), heparan sulphate (HS), keratin sulphate (KS) and hyaluronic acid (HA) are subtype sulphated polyssacharides of GAGs [12]. MPS, like other type of inherited metabolic disorders, is still remains under diagnosed by physician due to its rareness and complexity of symptom presentation [8].

Table 1 summarizes the demographic data of 58 patients included in this selective screening. This data is useful to understand the pattern of these patients as some of the patients showed prominent symptom of MPS however was found to be negative during screening and confirmation. Hepatomegaly and dysmorphic features are the most common symptoms among high risk patients during the study period. Clinicians and physicians would find this data useful for them to investigate any suspected MPS patients in future.

**Table 1:** High risk patients profiling of selective screening for mucopolysaccharidoses (MPS) in Malaysia (2014–2016).

No	Region	Age (Year)	Symptom	GAGs value (mmol/g creat)	GAGs level (based on age)	HRE band	Diagnosis
1	Northern	1.42	Hepatomegaly, Dysmorphic	53.3	Abnormal	Trace amount of DS band.	Normal
2	East Coast	2.00	Hepatomegaly, dysmorphic	35.34	Abnormal	Normal pattern	Normal
3	Northern	5.08	Dysmorphic, achondroplasia	15.27	Abnormal	Trace amount of HS band	Normal
4	Sarawak	6.00	Pectus carinatum, short stature	13.76	Normal	Normal pattern.	Normal
5	Sarawak	2.00	Pectus carinatum, short stature	18.55	Normal	Normal pattern.	Normal
6	Northern	5.00	Hepatomegaly, Dysmorphic	19.72	Abnormal	Normal pattern.	Normal
7	Northern	1.42	Hepatomegaly, Splenomegaly	34.97	Abnormal	Normal pattern.	Normal
8	Northern	56.00	Dysmorphic, short stature	6.86	Normal	Normal pattern.	Normal
9	Northern	16.00	Dysmorphic, short stature, kyphosis	5.36	Normal	Trace amount of HS band	Normal
10	East Coast	1.83	Dysmorphic, hepatosplenomegaly	28.02	Abnormal	Trace amount of HS band	Normal
11	Southern	9.00	Dysmorphic, Clawed hand, kyphoscoliosis	10.35	Normal	Normal pattern	Normal
12	Northern	0.25	Hepatomegaly, umbilical hernia	44.08	Abnormal	Normal pattern	Normal
13	East Coast	0.05	Dysmorphic	76.33	Abnormal	Increase of HS band	Normal
14	East Coast	4.92	Dysmorphic	1.84	Normal	Increase HS band	Normal
15	Sabah	2.25	Hepatomegaly, Dysmorphic	18.51	Normal	Normal pattern	Normal
16	East Coast	14.00	Eye lesions, dysmorphic	7.49	Normal	Trace amount of HS	Normal
17	Central	8.00	Joint contracture	11.89	Normal	Normal pattern	Normal
18	Sarawak	3.00	Hepatomegaly, Dysmorphic	9.61	Normal	Trace amount of HS band	Normal
19	Northern	0.17	Hepatomegaly, Dysmorphic	44.82	Abnormal	Normal pattern.	Normal
20	Central	4.00	Dysmorphic	18.54	Normal	Normal pattern.	Normal
21	Central	0.33	Hepatomegaly, Dysmorphic	34.82	Abnormal	Normal pattern	Normal
22	Central	0.50	Hepatomegaly, Dysmorphic	47.98	Abnormal	Normal pattern	Normal
23	Southern	12.06	Hepatomegaly, Dysmorphic	10.35	Normal	Trace amount of HS band	Normal
24	Central	4.00	Hepatomegaly, Dysmorphic	8.28	Normal	Normal pattern	Normal
25	Sabah	1.25	Dysmorphic, hepatosplenomegaly	16.72	Normal	Normal pattern	Normal
26	Central	2.00	Dysmorphic, hepatosplenomegaly	15.95	Normal	Trace amount of HS band	Normal
27	Northern	0.67	Dysmorphic, hepatosplenomegaly	42.36	Abnormal	Normal pattern	Normal
28	Southern	7.42	Dysmorphic, genu valgum/bowing legs	11.76	Normal	Marked increase in CS band	Normal
29	Northern	7.00	Kyphoscoliosis	92.82	Abnormal	Presence of HS band	Normal
30	Northern	10.00	Dysmorphic, hepatosplenomegaly	13.33	Abnormal	Presence of mild HS band	Normal
31	Northern	0.33	Hepatosplenomegaly, respiratory distress	29.91	Normal	Increase of CS band with presence of mild HS band	Normal
32	Northern	0.33	Corneal clouding	43.02	Abnormal	Mild increase of DS band and trace amount of HS band	Normal
33	Southern	6.00	Dysmorphic	7.93	Normal	Presence of trace amount of HS and DS band	Normal
34	Northern	14.00	Dysmorphic, short stature, kyphosis	3.81	Normal	Mild increase of HS band	Normal
35	Southern	0.02	Dysmorphic	40.92	Abnormal	Trace amount of HS band	Normal
36	Sabah	15.00	Dysmorphic, hepatosplenomegaly	53.41	Abnormal	Marked increase of HS band	Normal
37	Southern	11.42	Dysmorphic, Clawed hand	6.65	Normal	Increase of DS band and trace amount of HS band	Normal
38	Sabah	3.00	Dysmorphic, hepatosplenomegaly	19.7	Abnormal	Presence of trace HS band	Normal
39	Sarawak	0.16	Hepatosplenomegaly, Corneal clouding	67.76	Abnormal	Presence of HS band	Normal
40	Northern	1.58	Dysmorphic, gibbus, pectus carinatum	56.13	Abnormal	Presence of KS band	Normal
41	Central	3.00	Dysmorphic, respiratory problem	108.74	Abnormal	Increase of DS band and trace HS band	Normal
42	Northern	0.83	Dysmorphic, eye lesions	42.86	Abnormal	Presence of DS band and trace amount of HS band	Normal
43	Sarawak	4.00	Dysmorphic	76.28	Abnormal	Increase of DS and HS band	MPS I
44	Central	0.17	asymptomatic (sibling screening)	185.35	Abnormal	Increase DS and HS band	MPS II
45	Central	1.0	Dysmorphic, hepatosplenomegaly	–	–	(Result of GAGs screening and HRE characterization were performed in University Hospital)	MPS II
46	Central	2.50	Dysmorphic, hepatosplenomegaly	92.37	Abnormal	Presence of DS and HS band	MPS II
47	Central	2.00	Dysmorphic, Hepatomegaly	44.31	Abnormal	Presence of HS band	MPS IIIA
48	Central	1.58	Dysmorphic, Hepatomegaly	–	–	(Result of GAGs screening and HRE characterization were performed in University Hospital)	MPS IIIA
49	Central	3.12	Scoliosis, Claw hand	12.44	Normal	Normal pattern	MPS IVA
50	Central	7.00	Respiratory distress, dysmorphic	–	–	HS prominent (Result of GAGs screening were performed in University Hospital)	MPS IVA
51	Central	1.08	Dysmorphic, Hepatomegaly	27.64	Abnormal	Normal pattern	MPS VI
52	Central	7.33	Dysmorphic, Hepatomegaly	69.54	Abnormal	Presence of DS band with trace amount of HS band	MPS VI
53	Northern	3.25	Dysmorphic, corneal clouding	51.7	Abnormal	Presence of DS band	MPS VI
54	Central	0.18	Dysmorphic, respiratory problem	100.97	Abnormal	Normal pattern	MPS VI
55	Northern	2.83	Dysmorphic, macrocephaly	19.27	Abnormal	Normal pattern	MPS VI
56	Northern	2.83	Dysmorphic, macrocephaly	60.25	Abnormal	Normal pattern	MPS VI
57	Central	0.5	Dysmorphic, hepatosplenomegaly	28.41	Normal	Presence of HS band	MPS VI
58	Central	0.21	Dysmorphic, hepatosplenomegaly	39.75	Abnormal	Normal pattern.	MPS VI

Table 2 focuses on demographic data in study population. Male to female ratio is almost proportionate and eliminates gender bias. For ethnicity, we have divided the major races in Malaysia into 6 subcategories: Malay, Chinese, Indian, natives from Sabah and Sarawak region and indigenous people (Orang Asli). We also classified origin hospital of these patients into different regions. For instance, central region comprises any hospital in Federal Territory of Kuala Lumpur and Putrajaya, State of Selangor and State of Negeri Sembilan; northern region (State of Perak, Pulau Pinang, Kedah and Perlis); East Coast region (State of Kelantan, Terengganu and Pahang); Southern (State of Melaka and Johor); Sarawak region and Sabah region. We believe this demographic data would be useful among policy makers to decide whether selective screening should be conducted in selected regions or the whole country.

**Table 2:** Demographic data of high risk patients of selective screening for mucopolysaccharidoses (MPS) in Malaysia (2014–2016).

Variables	Frequency	Percentages
Gender		
• Male	31	53.4
• Female	27	46.6
Ethnicity		
• Malay	36	62.1
• Chinese	16	27.6
• Indian	2	3.4
• Sabah Native	1	1.7
• Sarawak Native	1	1.7
• Indigenous People (Orang Asli)	2	3.4
Region		
• Central	19	32.8
• Northern	20	34.5
• East Coast	4	6.9
• Southern	6	10.3
• Sarawak	5	8.6
• Sabah	4	6.9

Table 3 below describes distribution of GAGs among different age groups in study population. Each age group is carefully divided according to age group in GAGs determination. The data from this table supports the facts that GAGs distribution will be decreased towards increment of age.

**Table 3:** Glycosaminoglycans (GAGs) distribution between age group in high risk patients of selective screening for mucopolysaccharidoses (MPS) in Malaysia (2014–2016).

Parameters	Age group			
	Less than 1 year	1–4 years	4–9 years	More than 9 years
Median	43.02	19.13	12.83	7.49
Minimum value	29.91	8.28	1.84	3.81
Maximum value	76.33	108.74	92.82	53.41

In general, Table 4 below describes the diagnostic test evaluation for three different approaches in our selective screening of high risk patients of MPS in Malaysia. The first approach was using only GAGs determination using DMB method, the second approach utilised characterization of GAGs using High Resolutions of Electrophoresis (HRE) and the last approach was carried out using a combination of GAGs determination using DMB and GAGs characterization using HRE. The first approach showed high sensitivity but poor specificity while the second approach revealed high specificity but poor sensitivity. By using both analytical methods, we managed to achieve satisfactory performances of sensitivity and specificity (more than 80%). We believe this information will be beneficial to laboratory personnel in order to evaluate their performance and capabilities of current methods.

**Table 4:** Diagnostics test evaluation for distinctive approaches of selective screening for MPS in high risk patients in Malaysia (2014–2016).

Parameters	GAGs		HRE		Combination	
	Value	95% CI	Value	95% CI	Value	95% CI
Sensitivity (%)	81.25	54.35 to 95.95	45.00	23.06 to 68.47	87.50	61.65 to 98.45
Specificity (%)	47.62	32.00 to 63.58	81.58	65.67 to 92.26	83.33	68.64 to 93.03
Positive predictive value (%)	37.14	28.94 to 46.16	56.25	36.01 to 74.6	66.67	49.80 to 80.13
Negative predictive value (%)	86.96	69.61 to 95.10	73.81	64.84 to 81.16	94.59	82.62 to 98.47
Disease prevalence (%)	27.59	16.66 to 40.90	34.48	22.49 to 48.12	27.59	16.66 to 40.90

In conclusion, demographic data together with clinical symptoms/presentation and laboratory findings are important to assist clinician/researchers for future studies in MPS and can be employed to improve the diagnosis of MPS.

## 2 Experimental design, materials and methods

### 2.1 Study population and sample collection

This is a prospective cross-sectional study involving samples from high-risk children and young adults for MPS conducted over 2 years starting June 2014 to June 2016. A total of 58 urine samples for urinary GAGs quantitation and characterization and whole blood (n = 60) for enzymatic assays were received between 2014 and 2016. Urine samples (20 ml) were kept frozen while whole blood (6 ml) was processed to obtain plasma and leukocytes before stored at −80 °C. All the samples were collected from patients which had least two features of the following of inclusion criteria: (a) abnormal face features such as macrocephaly or coarse face; (b) corneal clouding or loss of visual acuity; (c) hearing impairment and recurrent middle ear infections; (d) recurrent respiratory tract infection; (e) valvular heart disease or heart murmur; (f) recurrent inguinal or umbilical hernia; (g) hepatosplenomegaly; (h) at least two symptom of musculoskeletal: (1) evolving joint contracture without obvious signs of inflammation, (2) joint laxity, (3) gibbus, (4) cervical spine stenosis and/or cord compression, (5) kyphosis or scoliosis, (6) pectus carinatum, (7) bilateral hip dysplasia, (8) progressive genu valgum after age of 3 years old, (9) short stature of unknown reason, (10) carpal tunnel syndrome. Patients presenting with mental retardation were excluded from this study.

### 2.2 Sample size calculation

$$\begin{array}{l} \text{n}=\frac{{\text{t}}^{2}\times \text{p }\left(1-\text{p}\right)}{{\text{m}}^{2}}\\ =\frac{{\left(1.645\right)}^{2}\times 0.0021\;\left(1-0.0021\right)}{{\left(0.01\right)}^{2}}\\ =\frac{2.706\times 0.00209559}{0.0001}\\ =\;56.7\text{ ∼ }57\text{ samples} \end{array}$$

Where,

n = required sample size

t = confidence level at 90% (standard value is 1.645)

p = estimated prevalence of mucopolysaccharidoses worldwide in percentage (1/48,780 × 100% = 0.0021) [7]

m = margin of error at 1% (0.01)

### 2.3 Quantitation and characterization of urinary GAGs

MPS urine test includes both quantitative analysis of total GAGs using dimethylmethylene blue method (DMB) and qualitative using High Resolution Electrophoresis (HRE). In brief, 30 μL of standards and patient samples were diluted with 120 μL of deionised water. 825 μL of freshly prepared DMB was later added and mixed thoroughly and analysed by spectrophotometer, at 520 nm. Standard graphs were plotted and used to calculate the value of GAGs in patients’ samples. Method is adapted from Nor A [5]. Equal amount of urine is added to cetylpyridinium chloride (CPC) buffer where GAGs in urine is precipitated to form a complex with CPC. The resulting CPC/GAGs complexes are dissociated by addition of lithium chloride and the GAGs re-precipitated with ethanol. The GAGs precipitate was re-dissolved in 20 μL of phenol red. Electrophoresis of the recovered GAGs was undertaken on cellulose acetate using divalent ion buffer system of high ionic strength (0.1 mol/L barium acetate). High resolution was achieved by making use of the different solubility of each GAGs in ethanol/buffer solutions of different concentrations. Interpretation is based upon the quantitative analysis of their relative amounts of excretion and pattern recognition of the specific sulphate(s) detected on HRE.

### 2.4 Enzyme activity in blood

Enzyme activity was performed in plasma or leukocytes. The leukocytes were extracted from EDTA blood by differential centrifugation as described by van Diggelen et al. (1990). Plasma and leukocyte pellets were kept frozen at −80 °C until analysis. The resulting leukocyte pellet was sonicated in ice for two 5-s bursts at 5 micro/amplitude followed by centrifuged. The supernatants were kept on ice before analysis. Modified Lowry method was used to determine protein concentration. For enzyme assays, methods from Lysosomal Laboratory of Willink Biochemical Genetics, St. Mary's Hospital, Manchester, UK were adapted with modification for the assays to be performed in microtiter plates. We used methods described by Hopwood et al. (1979) for MPS Type I [6], MPS II [14], MPS IVA [13], MPS IVB [4], MPS VI [2], and MPS VII [3]. Furthermore, various methods for determination of total β-hexosaminidase [9], β-mannosidase [10] and α-mannosidase [11] for diagnosis of mucolipidoses if all MPS enzyme assay were found normal.

Selected enzyme analysis was performed based on the qualitative results of HRE. Specified volumes of sample (plasma or leukocytes) were mixed with specific buffer and specific artificial substrates were tagged to a fluorescent compound, depending on the enzyme being assayed. The mixtures were incubated at specified times and the reactions were terminated by adding stop buffer. The enzymes in the sample were reacted with the artificial substrate and released the fluorescent compound. This compound was measured using fluorometer. Various concentrations of the fluorescence compound were run as standards and the curves were plotted and used for calculation of product amount. Enzyme activities in plasma were expressed as amount of fluorescent compound (product) being released per ml per hour (nmol/ml/hour). Enzyme activities in leukocytes were expressed as the amount of product being released per ml per mg protein per hour and the unit is nmol/ml/mg protein/hour.
